# Exome sequencing reveals genetic differentiation due to high-altitude adaptation in the Tibetan cashmere goat (*Capra hircus*)

**DOI:** 10.1186/s12864-016-2449-0

**Published:** 2016-02-18

**Authors:** Shen Song, Na Yao, Min Yang, Xuexue Liu, Kunzhe Dong, Qianjun Zhao, Yabin Pu, Xiaohong He, Weijun Guan, Ning Yang, Yuehui Ma, Lin Jiang

**Affiliations:** The Key Laboratory for Farm Animal Genetic Resources and Utilization of Ministry of Agriculture of China, Institute of Animal Science (IAS), Chinese Academy of Agricultural Sciences (CAAS), Beijing, 100193 China; Department of Animal Genetics and Breeding, China Agricultural University, Beijing, 100094 China

**Keywords:** Cashmere goat, Population genetics, High altitude, Hypoxia

## Abstract

**Background:**

The Tibetan cashmere goat (*Capra hircus*), one of the most ancient breeds in China, has historically been a critical source of meat and cashmere production for local farmers. To adapt to the high-altitude area, extremely harsh climate, and hypoxic environment that the Tibetan cashmere goat lives in, this goat has developed distinct phenotypic traits compared to lowland breeds. However, the genetic components underlying this phenotypic adaptation remain largely unknown.

**Results:**

We obtained 118,700 autosomal SNPs through exome sequencing of 330 cashmere goats located at a wide geographic range, including the Tibetan Plateau and low-altitude regions in China. The great majority of SNPs showed low genetic differentiation among populations; however, approximately 2-3 % of the loci showed more genetic differentiation than expected under a selectively neutral model. Together with a combined analysis of high- and low-altitude breeds, we revealed 339 genes potentially under high-altitude selection. Genes associated with cardiovascular system development were significantly enriched in our study. Among these genes, the most evident one was *endothelial PAS domain protein 1* (*EPAS1*), which has been previously reported to be involved in complex oxygen sensing and significantly associated with high-altitude adaptation of human, dog, and grey wolf. The missense mutation Q579L that we identified in *EPAS1*, which occurs next to the Hypoxia-Inducible Factor-1 (HIF-1) domain, was exclusively enriched in the high-altitude populations.

**Conclusions:**

Our study provides insights concerning the population variation in six different cashmere goat populations in China. The variants in cardiovascular system-related genes may explain the observed phenotypic adaptation of the Tibetan cashmere goat.

**Electronic supplementary material:**

The online version of this article (doi:10.1186/s12864-016-2449-0) contains supplementary material, which is available to authorized users.

## Background

Among the most severe environmental challenges to confront animals is the low oxygen availability of high-altitude regions such as the Tibetan Plateau, which is at an elevation above 5,000 m. The partial pressure of oxygen at 5,000 m is only approximately 50 % of the value at sea-level, and the resultant hypoxia imposes severe constraints on aerobic metabolism and leads to high-altitude illness [1–3]. Thus the mechanisms of hypoxic adaptation have become of great interest in recent years. Genome-wide scans and whole genome re-sequencing analysis for identifying selection signatures of high-altitude adaptation have been performed across a wide range of species, including human [4–6], dog [7, 8], grey wolf [9], yak [10], Tibetan antelope [11], cattle [12], pig [13], and chicken [14]. Although the convergent evolution between different species was reported in Tibetan people, dog and grey wolf with the *EPAS1* (*endothelial PAS domain protein 1*) gene as the common selected locus [4–9], in other species, distinct loci were revealed for high-altitude adaptation, suggesting that diverse molecular mechanisms may be employed.

Cashmere goat has the broadest altitudinal range of all herbivores in mainland China [15], as it is continuously distributed from sea level (e.g., Liaoning cashmere goat) to the Tibetan Plateau (e.g., Tibetan cashmere goat). As first recorded in 3,300-2,000 BC [16], the Tibetan cashmere goat exhibits phenotypic adaptation to the hypoxia conditions of the Tibetan Plateau under long-term natural and artificial selection [17]. Pioneering work regarding the high-altitude adaptation of the Tibetan cashmere goat mainly at the anatomical, biochemical, and physiological levels began two decades ago [18–23]. These early studies showed that the Tibetan goats and the F1 progeny of Tibetan goats × imported Liaoning cashmere goats (goats that originated from Liaoning and that are imported into Tibet) had stable inheritable adaptability to live in a high-altitude environment with some adaptive traits [24]. Compared with the goats living at an altitude below 3,000 m, the Tibetan goats maintained higher adult haemoglobin (Hb) concentrations, heavier heart and lungs, and lower heart rates [18–23]. Therefore, the Tibetan cashmere goat provides an outstanding model for understanding the genetic mechanism of hypoxia adaptation and hypoxia-related disease development. However, the molecular mechanism underlying the observed phenotypic adaptation to the high-altitude environment remains largely unknown.

Our previous studies regarding the Chinese cashmere goat, based on markers from both mitochondrial DNA and microsatellites [25–27], showed a high level of genetic differentiation between the Tibetan cashmere goat and other cashmere goat breeds [27]. Hence, we speculated that the Tibetan cashmere goat carries unique adaptive alleles for the high-altitude environment [27] that have never been identified due to the limited number of polymorphic markers. The development of next-generation sequencing and the release of the first goat reference genome assembly [28] offer us a great opportunity to investigate the genetic differentiation among goat breeds in a high-throughput method. Here, we extended our previous analyses with high-throughput sequencing of exome-captured genomic DNA to detect the genetic variation of six major Chinese cashmere goat populations (Table 1). We then conducted a genome-wide screen for the selection signatures at the exome regions in two high-altitude Tibetan cashmere goat populations (Bange [BG] and Ritu [RT]) by comparing these populations with the four lowland breeds (Chaidamu [CDM], Nanjiang [NJ], Inner Mongolia [IM], and Liaoning [LN]) to identify the possible molecular basis of the high-altitude adaptation (Table 1). This study is the first comprehensive analysis of the population genetic differentiation in Chinese indigenous cashmere goats and detection of the molecular mechanisms underlying the observed phenotypic adaptation of Tibetan cashmere goats.

**Table 1:** Information about samples collected from Chinese cashmere goats at different altitudes

Breed	Locality	Sample size		Altitude (m)	*P* ^a^	*H* ^b^
		F^♂^	M^♀^			
Highland						
Bange (BG)	Bange, Tibet	27	33	4,700	90.9	23.5
Ritu (RT)	Ritu, Tibet	31	37	4,500	86.9	22.9
Lowland						
Chaidamu (CDM)	Chaidamu, Qinghai	20	25	3,000	94.0	24.0
Nanjiang (NJ)	Aksu, Xinjiang	28	30	1,700	89.9	23.5
Inner Mongolia (IM)	Erlangshan, Inner Mongolia	14	25	1,500	88.8	23.2
Liaoning (LN)	Gaizhou, Liaoning	30	30	30	84.3	22.4

Note: P^a^: proportion of the total SNPs that have support for both alleles in each sample; H^b^: average heterozygosity = (sum of [2*p*(1-p)] for all SNP ) / (total number of SNPs), where p is the frequency of the most common allele; F^♂^: the sample size of female individuals; M^♀^: the sample size of female individuals

## Results

### Exome sequencing of the pooled samples

We sequenced six pools of captured exomes from goats living at different altitudes (Table 1) using a HiSeq 2000 platform, resulting in 50–84 million raw reads and 89- to 163-fold coverage for each pool (Additional file 1: Table S1). We aligned the reads to the reference genome of the domestic goat (version CHIR_1.0) and estimated the allele frequencies for all 144,046 SNPs identified by our SNP calling pipeline as described in the Methods section. In total, 118,700 autosomal SNPs were used in the downstream analysis. A strong correlation was observed between the sequence-based allele frequency and individual genotyping allele frequency estimates in each population (Pearson’s correlation test; *P*: *P* value; R: Pearson’s correlation coefficient; R_BG_ = 0.865, *P*_BG_ < 2.20E-16; R_RT_ = 0.933, *P*_RT_ < 2.20E-16; R_CDM_ = 0.903, *P*_CDM_ < 2.20E-16; R_NJ_ = 0.928, *P*_NJ_ = 2.20E-16; R_IM_ = 0.885, *P*_IM_ < 2.20E-16; R_LN_ = 0.872, *P*_LN_ < 2.20E-16; Additional file 2: Figure S1), indicating that our strategy of DNA pooling and exome capture was quite effective for estimating the allele frequency of the SNPs in the exome region at the population level.

### Genetic diversity analysis

**Fig. 1:**
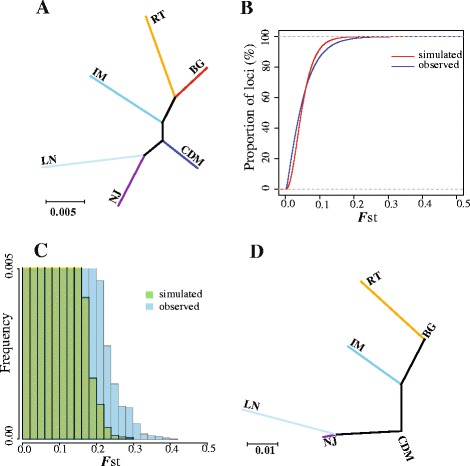
Phylogenetic analysis and *F*st simulation test. **a** Phylogenetic tree analysis based on all markers. **b** Cumulative distribution of observed and simulated (assuming neutrality) *F*st values. **c** Histogram of *F*st values in the simulated and observed datasets (note the truncated y-axis). **d** Phylogenetic tree based on those global SNPs showing significant genetic differentiation

The proportion of polymorphic loci and the average heterozygosity ranged from 84.3 % to 94.0 % and from 22.4 % to 24.0 %, respectively, with the lowest heterozygosity value in LN and the highest heterozygosity in CDM (Table 1), indicating the lowest diversity in LN and the highest in CDM. A phylogenetic tree based on all SNPs showed a clear separation among all populations (Fig. 1a). The topology structure of the tree was consistent with that of our previous result based on the mitochondrial DNA and microsatellite markers from the same cashmere breeds [25, 27]. The two Tibetan cashmere populations, BG and RT, formed one branch and differed greatly from other cashmere breeds. In the main branch of the lowland breeds, LN and NJ populations were grouped together, while CDM and IM were genetically distinct. Among all the branches of the tree, CDM had the shortest branch and LN had the longest one, which corresponded to the highest diversity in CDM and the lowest diversity in LN, consistent with the heterozygosity analysis (Table 1).

Because both natural selection and artificial selection have shaped the goat genomes and because genetic adaptations can occur due to many different variables, we first focused on the genetic differentiation in a global scenario to obtain a more comprehensive understanding of the selection patterns among all six populations. We estimated the global *F*st for each identified SNP, and this estimation was termed the global *F*st analysis. To evaluate the population diversity we calculated the average genetic differentiation coefficient (*F*st), the average overall genetic diversity (Ht) and the average diversity within a population (Hs), which were 0.0531, 0.2116 and 0.2007, respectively. This calculation indicated that only 5.31 % of the total genetic diversity was partitioned among breeds and that most of the variability (94.69 %) was within populations (Hs = 0.2007).

### Global *F*st analysis reveals regions under selection across six populations

To elucidate whether the observed genetic differentiation is primarily driven by drift or selection, a simulation test was conducted. The simulation was based on six subpopulations, each with an effective population size of 23,231 individuals who had been separated for 3,000 generations, and on the average simulated *F*st, which was identical to that observed in our global *F*st dataset (mean *F*st = 0.0531). Generally, the simulated data were consistent with the observed data (Fig. 1b). More than half (58.56 % and 55.15 % for the observed and simulated data, respectively) of the loci had *F*st values less than 0.05; however, a significant number of loci (*P* = 1.10E-16) had extreme *F*st values in the observed data compared with the simulation (Fig. 1c). Only 66 loci in the observed data had *F*st values greater than 0.2953, whereas none was found in the simulated data. Furthermore, 2.44 % of the loci in the observed data (*n* = 1,415) had *F*st values greater than 0.1723, whereas the corresponding value in the simulated data were 0.5 % (*n* = 290). Thus, approximately 2-3 % of the loci in our exome re-sequencing data showed greater genetic differentiation than expected under a selectively neutral model. Therefore, a threshold of the top 2.5 % was used to search for the loci under selection, and 1,449 SNPs, which overlapped with 880 genes, were found in the global *F*st analysis.

Gene Ontology (GO) analysis of the 880 genes revealed 33 significantly enriched GO categories. Among these categories, the most significantly enriched GO categories were cell adhesion (GO:0007155, *P_*FDR = 6.90E-06), regulation of molecular function (GO:0065009, *P_*FDR = 1.79E-06) and multicellular organismal development (GO:0007275, *P_*FDR = 4.69E-04) (Additional file 3: Table S2). We also found some interesting GO categories, such as response to external stimulus (GO:0009605, *P*_FDR = 2.20E-02), response to cell communication involved in cardiac conduction (GO:0086065, *P*_FDR =3.04E-2), blood vessel development (GO:0001568, *P*_FDR = 3.04E-02), and circulatory system process (*P*_FDR = 4.49E-02), which may be related to adaptation the local environment adaptation.

### Genetic differentiation between the high- and low-altitude goat breeds

**Fig. 2:**
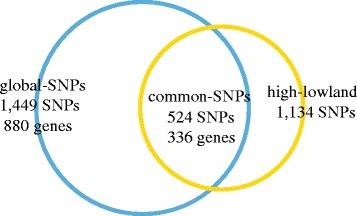
Overlap of SNPs from the global SNP dataset and high-lowland dataset

Given that the BG and RT populations have adapted to a high-altitude environment for thousands of years, we sought to identify the candidate loci adaptive to high altitude. The phylogenetic tree that was built from the top 2.5 % outlier SNPs of the global *F*st analysis showed a similar structure but with even clearer separation of the six goat populations (Fig. 1d) than the tree based on all SNPs (Fig. 1a). The new tree showed that the Tibetan cashmere goat populations (BG and RT) became even more divergent from other lowland breeds (Fig. 1d); however, the results were insufficient for determining whether the genetic differentiation resulted from high-altitude adaptation. Therefore, we evaluated the levels of population differentiation of BG and RT from other breeds to identify the loci showing evidence of adaption specific to the Tibetan goat using the statistic *di* [8] and χ^2^ test (see Methods section for details). By combining the *di* (di > 13.5) and χ^2^ test (FDR-adjusted *p* < 0.01) as the high-lowland analysis, we obtained 1,134 outlier SNPs that were genetically differentiated between highland and lowland goat populations (Additional file 4: Table S3; Fig. 2).

By comparing the candidate loci potentially under selection across all breeds to those genetically differentiated loci between highland and lowland populations, we identified 524 common SNPs that overlapped with 339 genes (termed as the common SNP dataset; Fig. 2; Additional file 5: Table S4). GO enrichment analysis with the 339 common genes showed a significant overrepresentation of the categories related to regulation of actin filament-based process (GO:0032970, *P*_FDR = 0.0266), cell adhesion (GO:0007155, *P*_FDR = 0.0365), regulation of molecular function (GO:0065009, *P*_FDR = 0.0465), regulation of multicellular organismal process (GO:0051239, *P*_FDR = 0.0465) and cardiac muscle cell action potential involved in contraction (GO:0086002, *P*_FDR = 0.0476; Table 2). Of the 339 common genes, 31 candidate genes were previously associated with high-altitude adaptation [9] and 66 were involved in cardiovascular system according to literature mining (Additional file 5: Table S4). Further protein-protein interaction network analysis showed that many hypoxia-related genes or cardiovascular system-related genes were observed in the protein interaction network (*P* = 8.83E-3) (Additional file 6: Figure S2). Based on the protein-protein interaction network, we identified that the ‘hub’ genes were hypoxia and cardiovascular system-related genes, including *sirtuin type 1* (*SIRT1*), *intercellular cell adhesion molecule-1* (*ICAM1*), *endothelin receptor type A* (*EDNRA*), *Yamaguchi sarcoma viral-related oncogene homolog 1* (*YES1*), and *plakoglobin* (*JUP*) (Additional file 6: Figure S2).

**Table 2:** Functional gene categories enriched for the common SNP dataset GO ID

	Term description	Number of genes	*P* value	FDR
GO:0032970	regulation of actin filament-based process	15	2.11E-06	0.0266
GO:0007155	cell adhesion	24	7.20E-06	0.0365
GO:0065009	regulation of molecular function	54	1.23E-05	0.0465
GO:0051239	regulation of multicellular organismal process	50	1.43E-05	0.0465
GO:0086002	cardiac muscle cell action potential involved in contraction	5	2.27E-05	0.0476

GO enrichment analysis was also conducted with the remaining 925 specific SNPs (601 genes) from the global SNP dataset only. Interestingly, categories that were associated with the cellular component organization and biological adhesion were still significantly enriched, but the categories related to cardiovascular system were not (Additional file 7: Table S5). This finding indicated that genes in the categories of cellular component organization and biological adhesion were under selection among all goat breeds, whereas genes related to cardiovascular system were under selection in the high-altitude goat breeds.

### The non-synonymous SNPs on *EPAS1* is associated with the high-altitude adaptation in a larger goat population

After filtering out genes with 120 LOC symbols, as many as 235 of 404 common SNPs were located at the protein-coding regions: 101, at introns; three, at upstream regions; 11, at untranslated regions (UTRs); and 54, at the intergenic regions. Among these 235 coding SNPs, 86 non-synonymous SNPs were found in 73 genes, with one to four SNPs per gene. Among these 73 candidate genes, four genes, *EPAS1*, *EDNRA*, *SIRT1* and *ryanodine receptor 1* (*RYR1*), are hypoxia-related genes according to a study by Zhang [9] (Additional file 5: Table S4). As many as 14 genes, including *EPAS1*, *EDNRA*, *SIRT1*, *RYR1*, *desmoglein 2* (*DSG2*), *cardiomyopathy associated 5* (*CMYA5)*, *receptor-type tyrosine-protein phosphatase eta* (*PTPRJ*), *FUT1*, *heart development protein with EGF-like domains 1* (*HEG1*), *tyrosine phosphatase receptor-type Z polypeptide 1* (*PTPRZ1*), *PAS domain-containing serine/threonine kinase* (*PASK*), *sialic acid-binding Ig-like lectin 1 sialoadhesin* (*SIGLEC1*), *Niemann-Pick disease type C1 gene-like 1* (*NPC1L1*) and *nestin* (*NES*), were previously reported to be associated with the cardiovascular system through literature mining (Additional file 5: Table S4). Non-synonymous amino acid replacements were assigned to categories according to changes in different physicochemical properties [29]. Of these genes, eight contain at least one relevant amino acid change that altered their physicochemical properties. These genes were *EPAS1*, *PTPRJ*, *EDNRA*, *FUT1*, *NES*, *NPC1L1*, *PASK*, and *PTPRZ1* (Additional file 5: Table S4; Additional file 6: Figure S2).

**Fig. 3:**
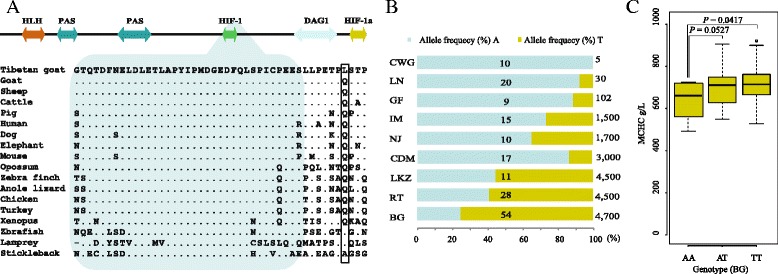
*EPAS1* mutation in the coding regions. **a** EPAS1 protein sequence analysis. The protein coordinate is based on NCBI RefSeq XP_005686651.1. The upper panel shows the Pfam domains of the protein. The double arrows represent domains of goat EPAS1. The orthologous protein sequences from 17 vertebrates are aligned with the mutant residues shown in the box. Sheep, ENSOARP00000006140; cattle, ENSBTAP00000004836; pig, ENSSSCP00000009011; human, ENSP00000406137; dog, ENSCAFP00000003819; elephant, ENSLAFP00000010336; mouse, ENSMUSP00000024954; opossum, ENSMODP00000001136; zebra finch, ENSTGUP00000004086; anole lizard, ENSACAP00000004025; turkey, ENSGACP00000015093; xenopus, ENSXETP00000031612; zebrafish, ENSDARP00000074832; lamprey, ENSPMAP00000000148; stickleback, ENSGACP00000015093. (HLH) helix-loop-helix domain; (PAS) Per-Arnt-Sim; (HIF) hypoxia-inducible factor; (CTAD) C-terminal transactivation domain; (DAG1) Dystroglycan (Dystrophin-associated glycoprotein 1), the blue shade represent the HIF-1 domain. **b** Percentages of the reference allele and variant allele in larger population samples that were genotyped with Sanger sequencing technology. The number on the bar represents the sample size. The altitude information is shown at the right side of the bars. **c** Association analysis between *EPAS1* genotypes (mutant allele, T; reference allele, A) and the MCHC in BG population. The ANOVA *F*-test was performed, and we found a significant association between the genotypes and MCHC

The *EPAS1* gene was the first gene that drew our attention because it was involved in complex oxygen sensing and was significantly associated with high-altitude adaption in dog and human [7, 8, 30]. This gene’s non-synonymous SNP (chr11: 28306765), which was identified as a potential target for selection at high altitude, causes a Gln 579-Leu (Q579L) mutation in the translated EPAS1 protein, based on GenBank annotation (Fig. 3a, Additional file 5: Table S4). The major allele of this SNP in high-altitude breeds is “T”, whereas the major allele in lowland breeds is the same as the reference allele “A”. Interestingly, the increasing frequency of the variant allele “T” with the elevated altitude was confirmed in the extended goat populations, with rare frequency in lowland breeds (Changjiangsnajiaozhou [CWG]: 0 %; LN; 7.5 %; Guangfeng [GF]; 11.11 %), moderate frequency in midland breeds (IM: 26.67 %; NJ: 35 %; CDM: 15.3 %), but high frequency in highland breeds (BG: 73.14 %; RT: 55.36 %; Langkazi [LKZ]: 59.09 %; Fig. 3b). In addition, this mutation occurred next to a well-defined protein domain (Hypoxia-Inducible Factor-1 domain, Fig. 3a). To further evaluate the functional significance of this variant, we aligned the mutant EPAS1 protein with its ortholog proteins in seventeen different vertebrates (Fig. 3a). The comparison revealed that Q579L mutated an evolutionary conserved amino acid, which is invariant among all the terrestrial animals that we examined. Statistical analysis of the blood test results in for the BG population revealed a statistically significant association between the genotype and the mean corpuscular Hb concentration (MCHC) (ANOVA *F*-test: *P* = 0.0417; Fig. 3c). All these results implied that *EPAS1* plays a critical role in high-altitude adaptation in the Chinese indigenous cashmere goat.

The second candidate gene is *PTPRJ* (also called *CD148* or *DEP1*), which is a member of the protein tyrosine phosphatase (PTP) family and which is present in all hematopoietic lineages [31]. Mutations in this gene can lead to defects in vascular development [32]. Six outlier SNPs were located in the *PTPRJ* gene; one in the 3’-UTR and five in the coding region. Of these five SNPs, four were non-synonymous, and three SNPs changed the physicochemical properties of the amino acid residues. However, multi-species alignment of the non-synonymous substitutions encoded in the *PTPRJ* genes showed that none of these four non-synonymous SNPs were present at evolutionary conserved sites. Surprisingly, the same type of SNPs occurred in the *NPC1L1*, *PTPRZ1, NES*, and *PASK* gene. For the EDNRA and FUT1, the non-synonymous substitutions were present at extremely evolutionary conserved sites; however, the variants in *EDNRA* and *FUT1* did not present increased allele frequencies in different goat populations as the altitude increased. Therefore, no further validation was conducted on these genes (Additional file 8: Figure S3).

## Discussion

### Genetic diversity among the Chinese cashmere goat populations

Using more than 100 K SNPs extracted from more than 300 goats of major cashmere breeds in China, an obvious separation across these breeds was observed. Phylogenetic analysis separated the Tibetan cashmere goat populations (BG and RT) from other groups. This result is consistent with previous reports from our own studies and other studies based on microsatellite and mitochondrial data [33, 34]. The other four Chinese cashmere goat breeds were grouped into three sub-clusters, with LN and NJ populations in one cluster and CDM and IM populations in two distinct branches (Table 1). These results are consistent with the breeding history of both CDM and NJ cashmere goats because they both originated from a cross between the local goat breeds (maternal) and LN (paternal). The genetic exchanges between LN and NJ, LN and CDM cashmere goat breeds for improving cashmere quality and quantity probably have led to a close relationship among these cashmere goat breeds.

We estimated the global *F*st value for each identified SNP; the average *F*st value was 0.0531, indicating that a small portion of the genetic diversity in the total population was attributed to differences among the populations. In addition, all six breeds had a high proportion of polymorphic loci and a similar average heterozygosity value, suggesting that a high level of variation has been maintained within these Chinese cashmere goat breeds. Our overall global *F*st value was slightly lower than those of previous studies of Chinese cashmere goats (0.063 [27]) and of other Chinese indigenous goats (0.056 [33], 0.054 [34]). The relatively lower *F*st value in our analysis can be explained by the fact that we used exome SNPs, which have a lower rate of substitution than intergenic SNPs.

### Population differentiation pattern of cashmere goat populations

The topological structure of the phylogenetic tree based on the top 2.5 % SNPs of the global *F*st analysis (Fig. 1d) slightly differed from that based on all SNPs (Fig. 1a), showing a relatively high level of genetic differentiation among all breeds (Fig. 1d). GO enrichment analysis showed that genes with remarkable genetic differentiation among breeds were enriched in many different categories (localization, cell adhesion and system development, and so on), thus indicating that the population differentiation pattern is complex and that the factor affecting the differentiation pattern is not unique. Both natural and artificial selection have contributed to the genetic differentiation in these cashmere goat breeds.

Since being subject to both artificial and natural selection for a long time, especially strong and directional selection recently, the evolutionary direction of the cashmere goat has been in line with human needs. China has a 2,500-year history of cashmere production: an estimated 10,000 tons of cashmere is produced per year [28]. Indeed, a few genes associated with cashmere traits displayed significant differentiation among the different goat breeds. For example, the global *F*st analysis indicated that the *Desmoglein 3* (*DSG3*) gene had the highest *F*st value (0.41) among the goat breeds; the gene plays an important role in maintaining the normal structure and function of hair [35]. GO categories such as skin development (*P* = 2.72E-2) may be relative to the differential cashmere production in these breeds (Additional file 3: Table S2).

In addition, many genes with high *F*st values in the global *F*st analysis play a role in the cardiovascular system. The GO categories previously reported to be associated with high-altitude adaptation in pig [36], such as blood vessel development, vasculature development, and cardiac conduction, were significantly enriched (*P* < 1E-4) in our analysis. The GO categories previously reported to be associated with high-altitude adaptation in grey wolf [9], such as response to external stimulus, were also significantly enriched in our study (*P* < 2.26E-02). Further analysis was performed to determine whether the above loci presenting significant genetic differentiation, such as cardiovascular system-related genes, are associated with high-altitude adaptation.

### Cardiovascular system-related genes play an important role in the high-altitude adaptation

Adaptation to a hypoxic environment manifests as a large phenotypic shift observed in the Tibetan cashmere goat compared with the goats living at low altitudes. Previous studies have showen that the Tibetan cashmere goat has a higher adult Hb concentration, a heavier heart, lung and trachea, lower heart rate, and a high rate of arrhythmia [18–20]. These adaptive phenotypic traits enable these animals to survive in the Tibetan Plateau. In the present study, we found molecular evidences in line with the phenotypic adaptations of the cardiovascular system.

First, genes related to cardiovascular system development were genetically differentiated in the global *F*st analysis (Additional files 3 and 7: Table S2 and Table S5, Table 2). Subsequently, we identified 66 (19.64 %) genes in the common SNP dataset that were previously identified as cardiovascular system development-related genes (Additional file 5: Table S4). High-altitude stresses include hypoxia, decreasing temperature, low humidity, and increasing ultraviolet radiation [37]; cold temperature and low oxygen are the two most remarkable factors that make survival at high-altitudes challenging [38]. Moving materials (especially oxygen) and regulating body temperature are the two most important functions of the cardiovascular system [39]. Thus, environment stresses of different altitudes are key factors that simulate the differentiation of goat population, and cardiovascular system-related genes may play an important role in adaptation to high-altitude environments in goats.

High-altitude adaptation may be due to multiple genes that act in concert with one another. Many cardiovascular system-related genes in the protein-protein interaction networks analysis are hub genes (Additional file 6: Figure S2) that are thought to play crucial roles in networks [40]. For example, *SIRT1* encodes a member of the sirtuin protein family, which can deacetylate and activate HIF2A in cultured cells subjected to hypoxia [41, 42]. *ICAM1* can be induced by hypoxia in aortic endothelial cells and cardiac myocytes [43–46]. EDNRA and YES1 were shown to be involved in high-altitude adaptation [5, 8, 47]; *EDNRA* encodes the receptor of endothelin-1, a peptide that plays a key role in potent and long-lasting vasoconstriction [48], whereas *YES1* encodes a protein with tyrosine kinase activity that belongs to the src family [49]. YES is realted to HSP27, which can be specifically upregulated through HIF1 signaling under the hypoxic conditions [50]. *JUP* encodes a major cytoplasmic protein that is the only known common component of the intracellular plaques of adhesions, predominantly desmosomes and adherens junctions [51]. Mutations in this gene have been associated with arrhythmogenic right ventricular cardiomyopathy [52, 53]. However, none of these hub genes except for *EDNRA* contained non-synonymous SNPs.

### Validation of *EPAS1* in a larger population

High-altitude human populations (Tibetans, Andeans, and Ethiopians) on different continents show distinct patterns of adaptation to high-altitude hypoxia [54], and different species on the same continent also show different patterns of adaptation to high-altitude hypoxia [55]. Tibetan cashmere goats display several key adaptive features for coping with the harsh environment at a high altitude. One of these features is that Tibetan cashmere goats display a higher than expected Hb concentration relative to their lowland counterparts and to acclimatized lowland goats. In contrast to Tibetan human and Tibetan pig populations, which show decreased Hb levels at high altitude, this feature is a crucial adaptation mechanism for goats that have adapted to a high-altitude environment [18]. The elevation of Hb at high altitude has also been observed in Tibetan yak and sheep [55].

The *EPAS1* gene is the most prominent candidate gene identified in this study. EPAS1, a member of the transcription factor family characterized by a basic helix-loop-helix (bHLH) and by a (Per-AhR-Arnt-Sim) PAS domain, plays a prominent role in process of adaptation to hypoxia and in the promotion of survival during hypoxic stress. *EPAS1* represents a “hot spot” of high-altitude adaptation research since several previously published studies provided evidence of an association between a few SNPs near or in *EPAS1* and the high-altitude adaption [8, 9, 12, 30, 56]. The *EPAS1* polymorphisms in the native Tibetan people were found to be associated with their lower Hb concentrations [30]. Several non-synonymous mutations on *EPAS1* have been identified in the Tibetan dog, including a key amino acid mutation thought to be associated with the lower blood flow resistance [7].

However, *EPAS1* is pleiotropic; therefore, other responses to hypoxia may be similarly affected. A few individuals living at low altitudes have been reported to have gain of function mutations in the HIF domains of EPAS1; these individuals exhibit excessive erythrocytosis and elevated Hb levels [57].

In our study, we also discovered a novel non-synonymous mutation, Q579L, on *EPAS1* in high-altitude goat populations; multi-species alignment indicated that this mutation is near to a well-defined domain (HIF) and is present at an evolutionary conserved site (Fig. 3a). Mutations in this domain of human EPAS1 are associated with erythrocytosis and elevated Hb levels [58, 59]. Goat EPAS1 Q579 corresponds to human EPAS1 Q557. A study in humans demonstrated that a novel mutation in human EPAS1 (Q557*) affects EPAS1 prolyl hydroxylation and VHL (von Hippel-Lindau tumor suppressor) protein binding, resulting in reduced EPAS1 ubiquitination but intact transcriptional activity and activation activity [60]. Further screen of this allele in additional Tibetan and lowland goat populations further confirmed a large divergence between low- and high-altitude goat populations (Fig. 3b). Furthermore, a statistically significant association between the genotypes and MCHC was found (Fig. 3c), implying the biological relevance of the mutant allele in the elevated MCHC levels of the Tibetan cashmere goat.

Taken together, these results indicate that EPAS1 may play a key role in the adaptation of Tibetan cashmere goats to a high-altitude environment at the molecular level; however, the adaptation patterns may not as the same in human and dog.

## Conclusions

Our study provides insights concerning the high-altitude adaptation of the important goat breeds in China, which is crucial for the sustainable utilization of cashmere goat. A high level of variation has been maintained within the Chinese cashmere goat breeds. Genes involved in the cardiovascular system development and the missense mutation of *EPAS1* may play a crucial role in the high-altitude adaptation of Tibetan cashmere goats.

## Methods

### Sample collection and preparation

Genomic DNA was isolated from ear tissue of 330 goats collected from six locations (LN, 30 m; IM, 1500 m; NJ, 1700 m; CDM, 3000 m; RT, 4500 m, BG, 4700 m, Table 1). The DNA isolated from each individual per sample location was quantified using a Nanodrop 2000 (Thermo Fisher Scientific, DE) and pooled in equimolar concentrations.

The peripheral blood samples from 60 BG cashmere goats (Tibetan goats) were collected and tested individually at Tibet Autonomous Region People’s Hospital within 24 hours using a Sysmex XE-2100 Autoanalyzer (Sysmex Corporation, Kobe, Japan). Hb and MCHC values were measured by blood tests. The 60 BG cashmere goats whose Hb levels were measured were the same goats used in the exome re-sequencing.

The validation in the larger population included the following samples: Tibetan goat (BG, 54; RT, 28; LKZ, 11), CDM cashmere goat (17), NJ cashmere goat (10), IM cashmere goat (17), GF goat (9), LN cashmere goat (20), and CWG goat (10). All animal experiments in this study were fully approved by the Animal Care and Use Committee of the Ministry of Agriculture of the People’s Republic of China.

### Exome sequencing and reads mapping

For each DNA pool, a targeted exome library with an insert size of 150–200 bp was constructed by an exome capture strategy, using a GenCap custom exome enrichment kit (MyGenostics, Beijing, China). An Illumina HiSeq 2000 platform was used to generate paired-end 100-bp raw reads for each enriched library according to the manufacturer’s protocol. Quality filtering of the raw reads was performed using NGSQC Tookit v2.3.3 [61]. For each sample, we discarded reads that did not have an overall quality score of 20 using the PHRED33 scale for at least 70 % of the bases or for any base with quality less than 20. Only paired reads were used for further analysis. The final depth of coverage of the six populations was between 83-155X.

### SNP detection

After removing low quality reads, we used BWA (v0.7.10) [62] software with the default setting to separately map the paired-end reads that passed quality filtration separately from each of the six populations (Table 1) to the goat reference genome assembly CHIR_1.0 [28]. Duplicated reads were removed and mapping statistics were calculated using the SAMtools package (v0.1.19) [62]. Then, the SNPs were called using the SAMtools pipeline on a per-population basis [63]. We filtered the resulting SNPs using the following steps: 1) positions with read coverage lager than 200 were filtered to avoid calling SNPs in parts of the exome with excess coverage, likely the duplicated regions; 2) SNP positions with a read depth of at least 60 in the union of all reads from all populations were kept for further analysis; and 3) the positions that had less than 10 supporting reads for the variant allele were removed. The major SNPs from the BG population were selected as reference SNPs. ANNOVAR (v2014-07-22) was used to annotate the variants after filtering [64]. We validated the sequence-based allele frequency estimates by individual genotyping of the same goats for pooled sequencing. A total of 53 SNPs were selected for the validation and all were successfully genotyped in the six goat populations (Additional file 9: Table S6).

### Genetic differentiation analysis across populations using global *F*st values

We estimated the allele frequency of each SNP site for each population by analyzing read count data for the sequencing pool. For each SNP site that had a minimum of 30 reads from each of the six sampled breeds, global *F*st values across six populations were calculated using the method from Nei (1987, p.191) [65]. To describe the expected sampling distribution of *F*st values under a perfect neutral model, a simulation was conducted using the POWSIM software [66]. This program mimics a large base population segregating for a specified number of independent, selectively neutral loci with defined allele frequencies under the Wright-Fisher model, and then split the base population into *s* subpopulations of equal effective size (*Ne*) through random sampling of 2*Ne* genes. After drift for *t* generations for each subpopulation, the expected degree of divergence is *F*st = 1 - (1- 1/2*Ne*)^*t*^ [65].

To reduce the sampling bias of *F*st, 57,948 SNPs with the minimum coverage ≥ 30 for each of the six sampled populations were used to perform the simulation. Over all these SNPs, the average frequency of the most common allele was ~0.8619, and the average *F*st was 0.0531.

The objective evidence from archaeological excavations and historical records demonstrated that Tibetan cashmere goat was first recorded at Chamdo County in Tibet and has resided in the plateau area since 4,000-5,000 years ago [16, 67, 68]. In addition, China has a 2,500-year history of cashmere production [28, 69]. However, the effective population sizes of the base sampled populations are unknown, and as discussed in a study by Sangeet [70], exact values of *Ne* have little effect on *F*st distribution [70]. Moreover, for the bezoar, the ancestor of goat, females become sexually mature at 2–3 years [71], while for the Chinese cashmere goats, females become sexually mature at 150–180 days [68, 72]. Therefore, we set the generation time to one year and the *t* to 3000 generations for the Chinese cashmere goat. According to the above-mentioned information and formula, we tested various combinations of *Ne* and *t* to reach *F*st ≈ 0.0531 and simulated a relative large base population where a single biallelic locus segregated at a frequency of 0.8619. The base population was split into six subpopulations of effective size *Ne* = 23,231, and then these subpopulations were allowed to drift apart for *t* = 3,000 generations to arrive at an expected *F*st = 0.0531 for neutral loci, with 1 allele sampled from each subpopulation. If we had used *Ne* = 23,231 and *t* = 3,000, the simulation would take a long time (several weeks); therefore, we changed the *Ne* and *t* values to 2,749 and 300, respectively. These values can produce almost an identical *F*st distribution. After *F*st was calculated and this process was repeated 57,948 times, we compared the distribution of simulated *F*st values with the observed values. The largest simulated *F*st value was *F*st =0.2953, and the SNPs exhibiting *F*st > 0.2953 were considered under directional selection.

### Genetic differentiation between highland and lowland populations

To define the candidate positions that have undergone directional selection during high-altitude adaption, 57,948 SNPs with minimum coverage ≥ 30 for each of the six populations were used in the high-lowland *F*st analysis. Six goat populations were divided into two groups based on the altitude; the highland group (BG + RT) consisted of goat populations originating from Tibet, and the lowland group (CDM + NJ + IM + LN) consisted of goat populations originating from other locations. To retrieve candidate SNPs under selection in highland breeds, the *di* values, which combined the *F*st value for each breed were calculated for each SNP as described in Akey *et al*. (2010). A general statistic *di* [73] was based on all eight comparisons between the highland and lowland groups. *χ*^*2*^ test [70] was used to compare the allelic frequency between highland and lowland breeds. We performed a χ^2^ test, as a second empirical outlier analysis, combining the highland population group (BG + RT) as the case population and treating the other lowland population group (CDM + NJ + IM + LN) as a control group. In total, 1,449 SNPs that were within the top 2.5 % of the empirical distribution were identified in each analysis. Due to a smaller effective population size than the autosomes, X chromosomal and unplaced regions were not included in the analysis. The complete workflow of the data analysis is presented in Additional file 10; Figure S4.

### Phylogeny analysis

Based on the genetic distance matrix that was generated from the population allele frequency data of SNPs in the six populations, the neighbor-joining phylogenetic trees was constructed using POPTREE software [74]. The phylogenetic trees were displayed by TreeView [75].

### Candidate SNPs genotyping in a large population and *EPAS1* mutation analysis

The primers for polymerase chain reaction (PCR) were designed for genotyping of the individual goat to measure the accuracy of the site frequency survey using a pooled strategy. After PCR amplification, Sanger sequencing technology was employed to verify the target SNPs in ten different populations.

The protein domains of EPAS1 were predicted by the Pfam web service [76]. The full-length amino acid sequences of EPAS1 in 17 representative vertebrates species were retrieved from Ensembl revision 78, and only one-to-one orthologs were included (Fig. 3). A non-synonymous mutation of interest at *EPAS1* (chr11: 28306765) was genotyped on a larger population using traditional Sanger sequencing technology. All PCR primers used in this study are listed in Additional file 9: Table S6.

### Enrichment analysis

GO categories and function association networks were defined with the STRING v9.1 program using standard parameters [77], and removal of the redundant GO terms from this list was performed with REVIGO using standard parameters [78]. In all tests, the whole set of autosomal genes was appointed as the background and FDR adjusted *P* values, which indicate the significance of the overlap between various gene sets, were calculated. Only the terms with a FDR-adjusted *P* value < 0.05 were considered statistically significant and were listed. Genes with LOC symbols were not used in the enrichment analysis.

### Data access

All raw sequencing data from this study have been deposited in the Sequence Read Archive (SRA) database at the NCBI under accession number: SRX1009748, SRX1013181, SRX1013324, SRX1013922, SRX1013947, and SRX1013952.

## Additional files

Additional file 1: Table S1. Whole exome sequencing results for six goat populations. (XLSX 11 kb) Additional file 2: Figure S1. Positive correlation between site frequencies using a pooling strategy and Sanger sequencing technology for Tibetan Bange cashmere goat (BG, a), Tibetan Ritu cashmere goat (RT, b), Chaidamu cashmere goat (CDM, c), Nanjiang cashmere goat (NJ, d), Inner Mongolia cashmere goat (IM, e), and Liaoning cashmere goat (LN, f). (PDF 197 kb) Additional file 3: Table S2. Functional gene categories enriched for the global SNP dataset. (XLSX 12 kb) Additional file 4: Table S3. Outlier SNPs genetically differentiated between highland and lowland goat populations. (XLS 214 kb) Additional file 5: Table S4. SNPs in common SNP dataset. Notes: The amino acids physicochemical properties involved in the non-synonymous mutations were characterized according to the definition provided in the study by Moilanen JS and Majamaa K [29]. Green denotes genes related to high-altitude adaptation that were listed in the study by Zhang [9]; grey denotes genes related to hypoxia and the cardiovascular system in GO categories; yellow denotes genes related to the cardiovascular system according to the literature. (XLSX 87 kb) Additional file 6: Figure S2. Protein-protein interaction network with the common SNP dataset. Notes: Proteins without connections were removed for clarity. Red circle, proteins related to hypoxia and the cardiovascular system from the GO categories; yellow circle, proteins related to the cardiovascular system from the literature; green dot, proteins related to hypoxia adaptation listed in a study by Zhang [9]. “line”: interaction with medium confidence; “bold line”: interaction with high confidence. (PDF 405 kb) Additional file 7: Table S5. Functional gene categories enriched for the global SNP specific dataset. (XLSX 10 kb) Additional file 8: Figure S3. The multi-species alignment of non-synonymous substitutions encoded in the *NPC1L1*, *NES*, *PTPRJ*, *PTPRZ1*, *FUT1*, *EDNRA*, and *PASK* genes. (PDF 332 kb) Additional file 9: Table S6. Primer and SNP information for verification using Sanger technology. (XLSX 13 kb) Additional file 10: Figure S4. Workflow of data analysis. (PDF 352 kb)

## Abbreviations

*EPAS1*: *Endothelial PAS domain protein 1*
GO: Gene ontology
*SIRT1*: *Sirtuin type 1*
*ICAM1*: *Intercellular cell adhesion molecule-1*
*EDNRA*: *Endothelin receptor type A*
*YES1*: *Yamaguchi sarcoma viral related oncogene homolog 1*
*JUP*: *Plakoglobin*
UTR: Untranslated region
*RYR1*: *Ryanodine receptor 1*
*DSG2*: *Desmoglein 2*
*CMYA5*: *Cardiomyopathy associated 5*
*PTPRJ*: R*eceptor-type tyrosine-protein phosphatase eta*
*HEG1*: *Heart development protein with EGF-like domains 1*
*PTPRZ1*: *Tyrosine phosphatase receptor-type Z Polypeptide 1*
*PASK*: *PAS domain containing serine/threonine kinase*
*SIGLEC1*: *Sialic acid binding Ig-like lectin 1 sialoadhesin*
*NPC1L1*: *Niemann-Pick disease type C1 gene-like 1*
*NES*: *Nestin*
MCHC: Mean corpuscular haemoglobin concentration
*DSG3*: *Desmoglein 3*
VHL: Von Hippel-Lindau tumor suppressor
