# Supplementary figures and images for: Exome sequencing reveals genetic differentiation due to high-altitude adaptation in the Tibetan cashmere goat (Capra hircus)
Source: BMC Genomics. 2016 Feb 18;17:122. doi: 10.1186/s12864-016-2449-0 (PMC4758086; doi:10.1186/s12864-016-2449-0)

Figure S1

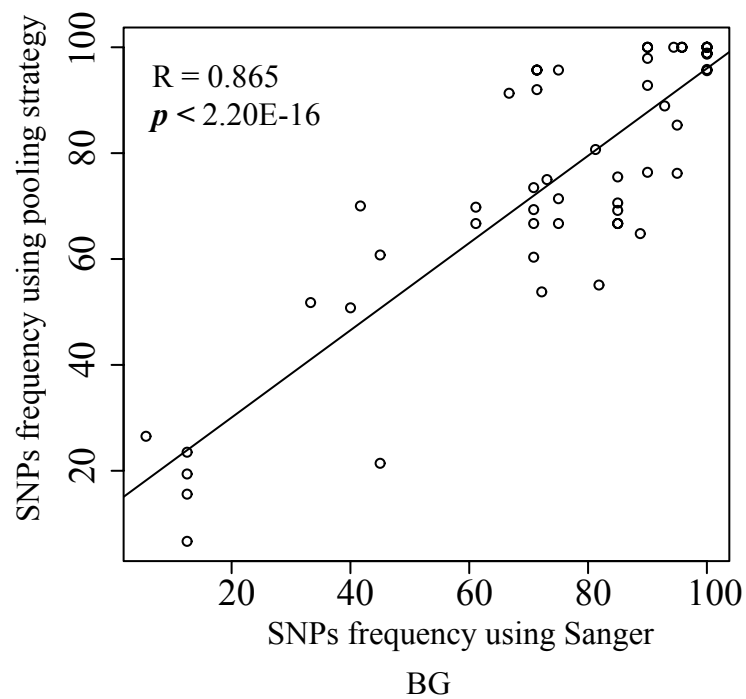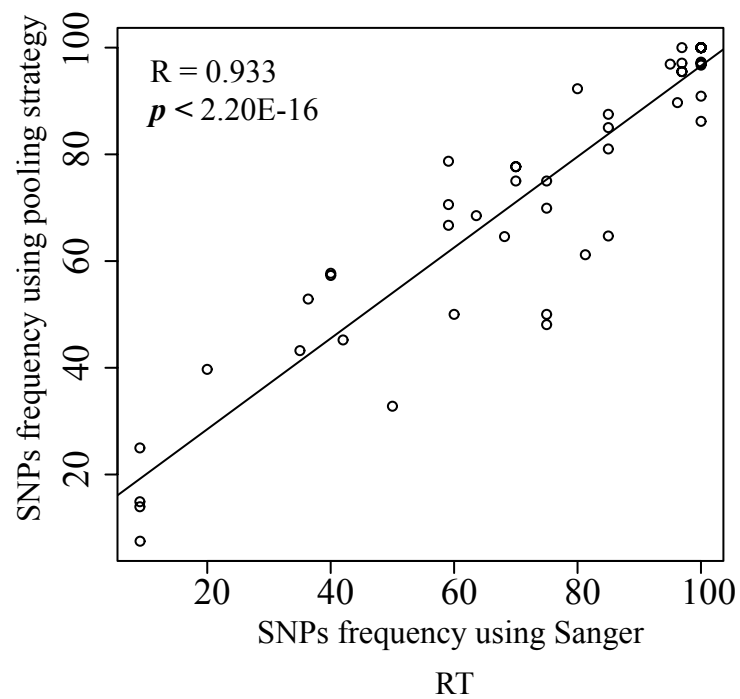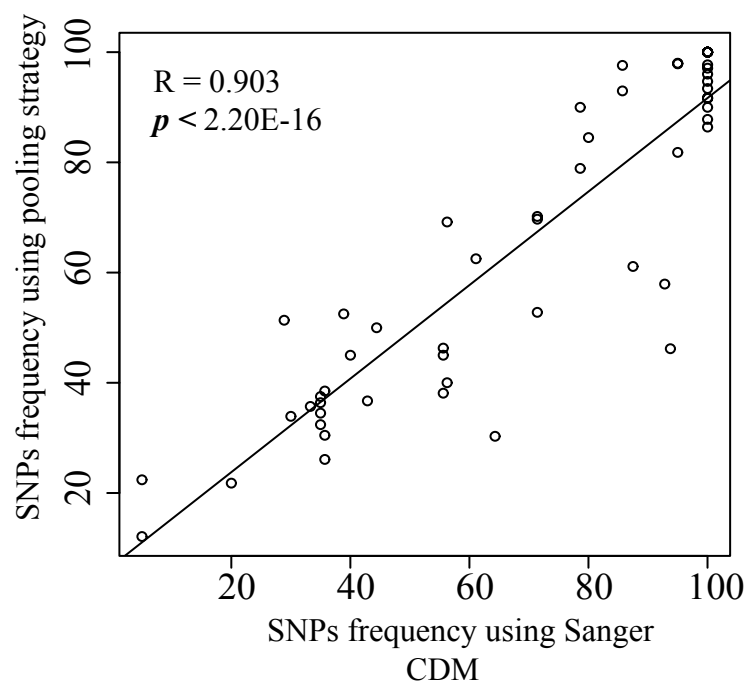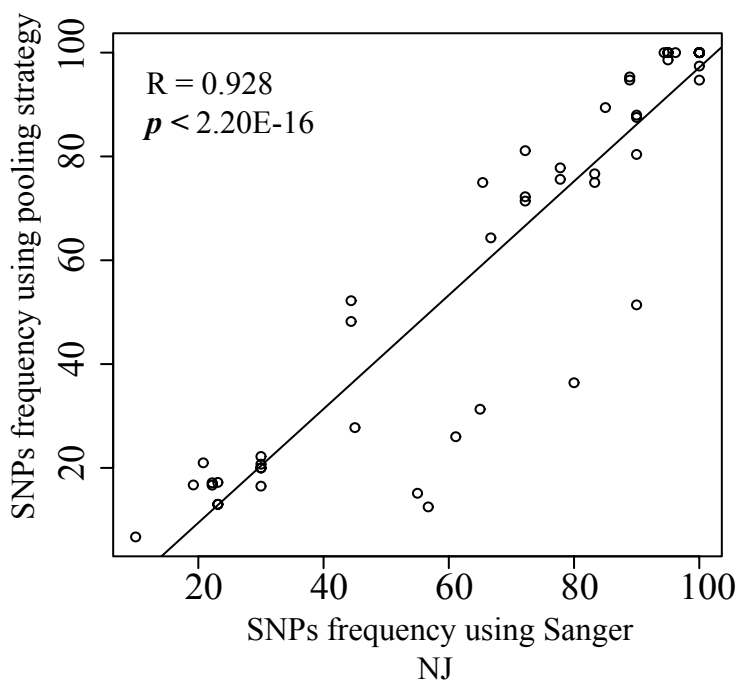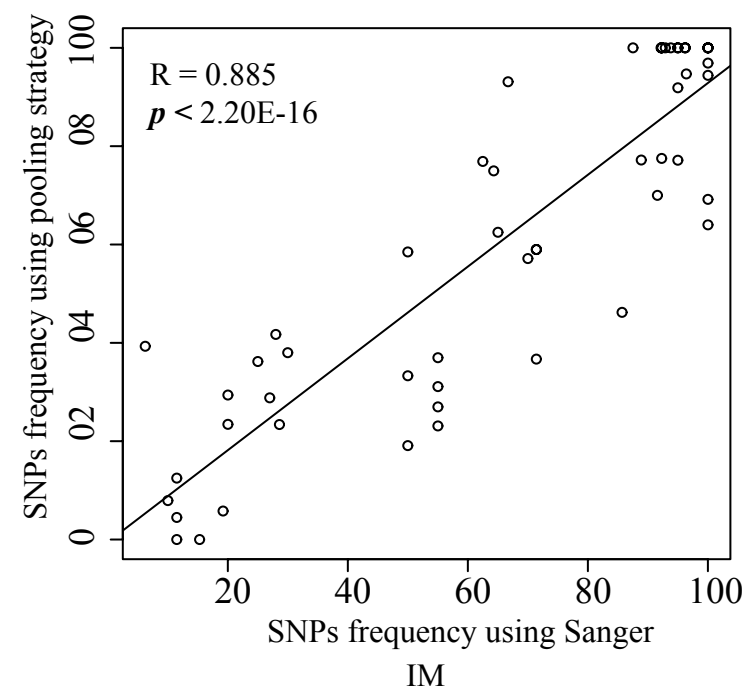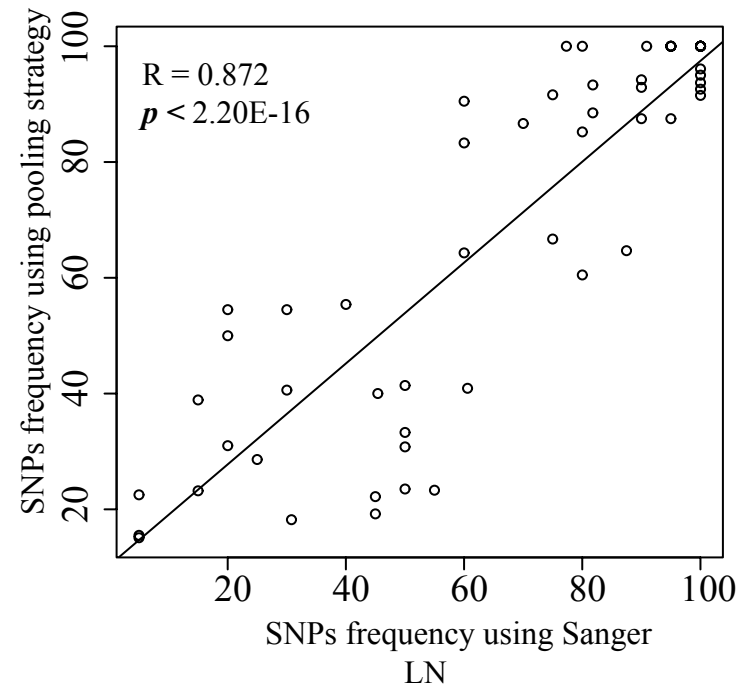

Supplement: Additional file 2: Figure S1. — Positive correlation between site frequencies using a pooling strategy and Sanger sequencing technology for Tibetan Bange cashmere goat (BG, a), Tibetan Ritu cashmere goat (RT, b), Chaidamu cashmere goat (CDM, c), Nanjiang cashmere goat (NJ, d), Inner Mongolia cashmere goat (IM, e), and Liaoning cashmere goat (LN, f). (PDF 197 kb) [file 12864_2016_2449_MOESM2_ESM.pdf]

Figure S2

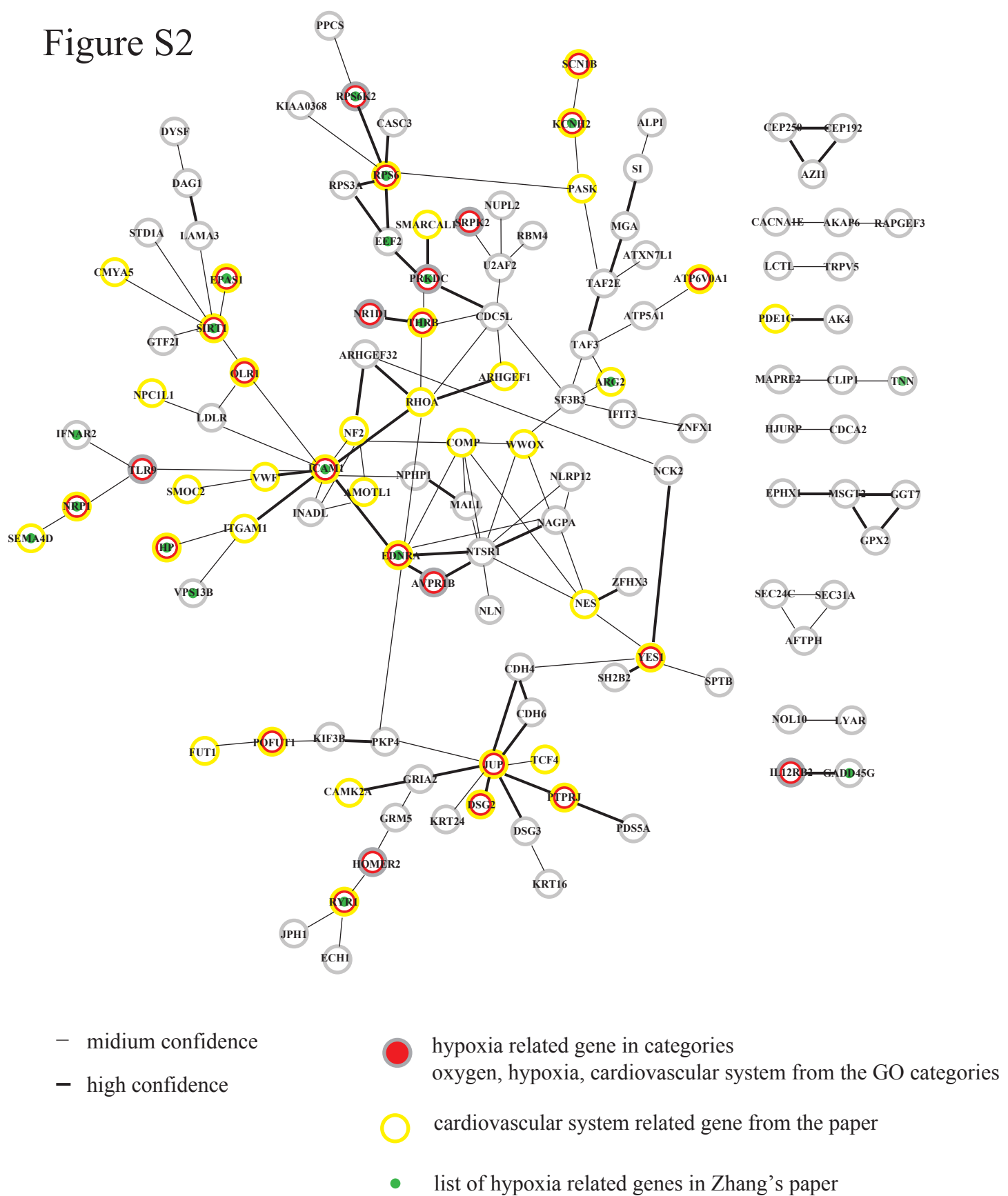

Supplement: Additional file 6: Figure S2. — Protein-protein interaction network with the common SNP dataset. Notes: Proteins without connections were removed for clarity. Red circle, proteins related to hypoxia and the cardiovascular system from the GO categories; yellow circle, proteins related to the cardiovascular system from the literature; green dot, proteins related to hypoxia adaptation listed in a study by Zhang [9]. “line”: interaction with medium confidence; “bold line”: interaction with high confidence. (PDF 405 kb) [file 12864_2016_2449_MOESM6_ESM.pdf]

Figure S4

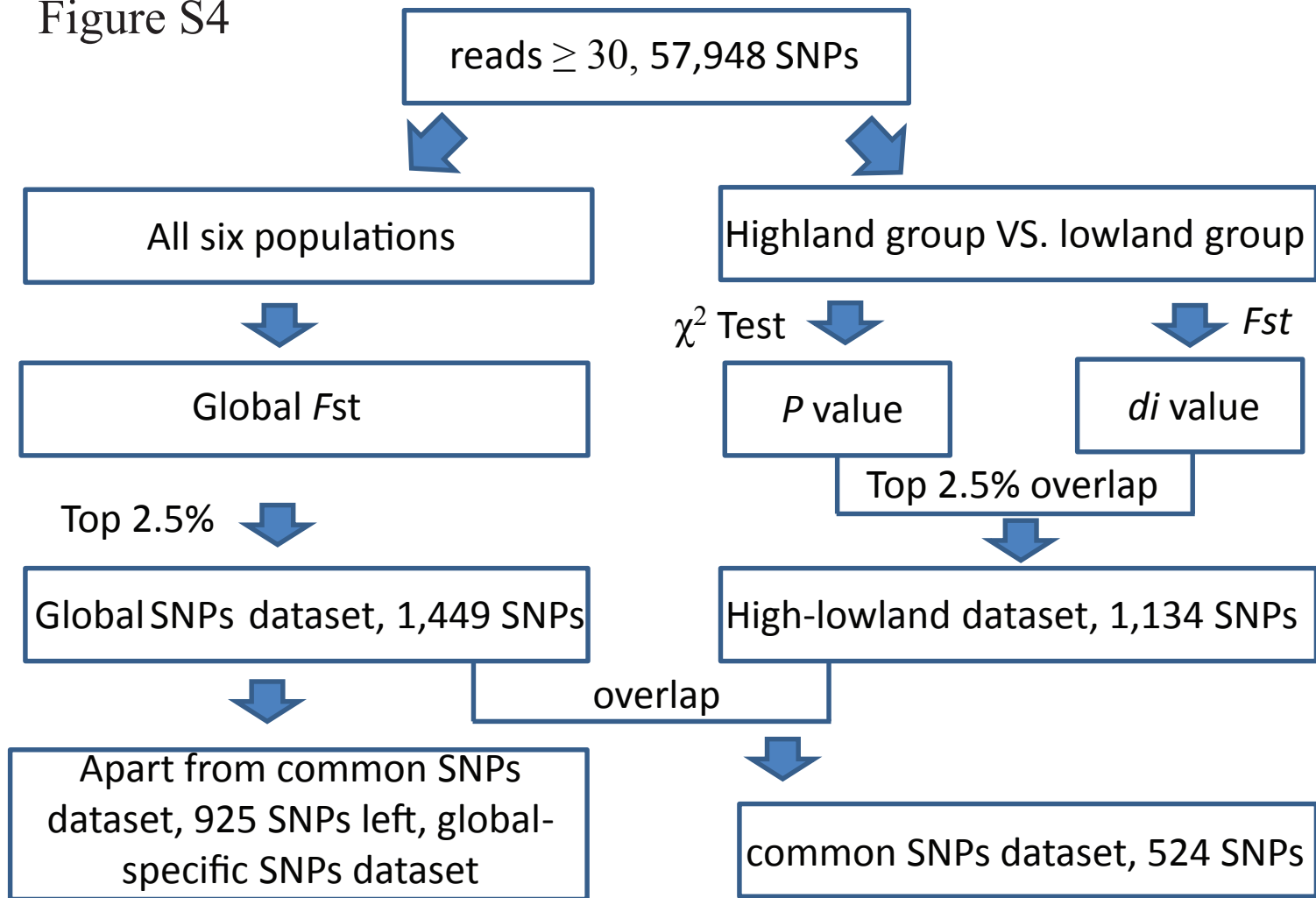

Supplement: Additional file 10: Figure S4. — Workflow of data analysis. (PDF 352 kb) [file 12864_2016_2449_MOESM10_ESM.pdf]
