# Supplementary material for: Exome sequencing reveals genetic differentiation due to high-altitude adaptation in the Tibetan cashmere goat (Capra hircus)
Source: BMC Genomics. 2016 Feb 18;17:122. doi: 10.1186/s12864-016-2449-0 (PMC4758086; doi:10.1186/s12864-016-2449-0)
Supplement: Additional file 8: Figure S3. — The multi-species alignment of non-synonymous substitutions encoded in the NPC1L1, NES, PTPRJ, PTPRZ1, FUT1, EDNRA, and PASK genes. (PDF 332 kb) [file 12864_2016_2449_MOESM8_ESM.pdf]

Figure S3

NPC1L1

```
             226        232        242
Tibetan goat PS---QAEGRVIQPLNDEVV
Goat         ..---....S..........
Sheep        ..---....S..........
Cattle       ..---....ST.........
Pig          ..---..A.S......G...
Human        .G---..V.SG.....EG.A
Elephant     ..---..P.NG..L..S.I.
Mouse        .G---..LADGMK..DGKIT
Opossum      .G---.VL.NGLE..DV.TW
Xenopus      SN---TEI.HG.V.HYSRVW
Zebrafish    D.E-TSEVPAGAFAFAGQAL
Lamprey      DGETAPQ.SQGMRQF..TTF
Stickleback  EGQ-TEGLPKGVI.Y.GRAL
```

NES

```
             897        907        917
Tibetan goat QSLEEADKGSLRHLEEEEIVE
Goat         ..........R..........
Sheep        ......A.....R......L.
Cattle       ............L....D..
Pig          R.P..EG.E.Q.YL.A..N..
Human        R.P..V..E.Q.NL....NLG
Dog          R...KENQEPV.SLV.D----
Elepant      K.S..V..E.Q.YL...GNL.
Mouse        E.PGGVE-D.QQCL.V..GP.
Opossum      ENQ.IPE--------.GKGAL.
Chicken      EVHGDQQEEHGDLQ.GNGDIQ
Xenopus      EEQSNSETEDNIGL.Q.CDL.
```

PTPRJ

```
             137   143  148     170                182  187 283 288  292
Tibetan goat DLKLGSRYTITL  ----AGNTDSLPG---GPATRGSKYR  TDSFNFTVNE
Goat         ......Q.....  ----.S.........---....P.....  .....L....
Sheep        ...M..H..V..  ----...........---...TP.....  .....L....
Pig          ...P.I..KV..  ----.N.....SQPLRS.GVPGY...  .....L....
Human        G..P.VQ.N.NP  ----....ERSRAG--S.TAPVHDES  ...S.LN.S.
Dog          G.IP.T.NKF..  ----.NS.E.SSQQSTT.TDSV.QSD  .G.L.LS..T
Elephant     V..P.TQ.WF..  ----.N..ERS..E--K.PQ-----N  .R...L.A.V
Mouse        G..P.TNNSFAF  ----.NG.KRI.VT---NLSQLH.NS  .H.V.Q...K
Opossum      IWH..VCQA.IY  ----SMVD....AA-FD.STSTH.CN  GH..DQNS.V
Zebra finch  G.IP.TP.NF.V  AETIGVTSVT.KWGVDDS.SDSYL..  ------.NDT
Anole lizard N.NP.TS..FRV  AENISNT.VT.KWDRND.DASNYT.Q  SA.WSEFS.N
Chicken      G.IP.TL.SFKV  AEYVGVTSVN.TWTVN.T.LTAYT..  SSIN.K.SDI
Turkey       G.MP.TL.SFKV  ------------------------C.  LSIN.E.SDI
```

PTPRZ1

```
             1134     1143  1149
Tibetan goat QSSHAVSQASGDTSLK
Goat         .........F......
Sheep        .........F......
Cattle       .........F......
Pig          ....T....F......
Dog          .V...LAPGGRRAPAT
Elephant     .PT.....TF...L..
Mouse        .PT.TA.......W..
Opossum      .PL..T.P.F...L..
Zebra finch  .PL.VT.P..D..L.R
Anole lizard .PL.VTTP.FD..L..
Turkey       .PL.VTTP.FD..L..
Xenopus      KQINTLPTTSAN.S.T
```

EDNRA

```
             120      129      138
Tibetan goat ASLALGDLIHVVIDLPINV
Goat         .........Y.........
Sheep        .........Y.........
Cattle       .........Y.........
Human        .........Y.........
Dog          .........Y.........
Elephant     .........Y.........
Mouse        .........Y.........
Oppssum      .........YI...I....
Zebra finch  .........YI...I..I.
Anole lizard ....M....YII..I....
Chicken      .........YI...I..I.
Turkey       .........YI...I..I.
Xenopus      .........YI...I..I.
```

FUT1

```
             145   151    158
Tibetan goat ELHDWMLEEYAHVK
Goat         ......S.......
Sheep        ......S.....L.
Cattle       ......S.....L.
Pig          ......S.D...L.
Human        Q.....S....DLR
Elephant     K.....S.....L.
Mouse        V.....S...S.LE
Opossum      P.R...A....QLG
```

PASK

```
             1317      1327      1336
Tibetan goat SDVHVNELLSIQPGTHPQQM
Goat         ..........T.........
Sheep        ....................
Cattle       ....................
Pig          ....AH...RL....R...L
Human        .NA.I.....VRR.ASL..L
Elephant     ..A.IR........RPT..L
Mouse        AEA.IHS.F.I..SV....L
```
